# Communicating incidental and reportable findings from research MRIs: considering factors beyond the findings in an underrepresented pediatric population

**DOI:** 10.1186/s12874-021-01459-8

**Published:** 2021-12-05

**Authors:** Kiley B. Vander Wyst, Micah L. Olson, Smita S. Bailey, Ana Martinez Valencia, Armando Peña, Jeffrey Miller, Mitchell Shub, Lee Seabrooke, Janiel Pimentel, Kiri Olsen, Robert B. Rosenberg, Gabriel Q. Shaibi

**Affiliations:** 1 College of Graduate Studies, Midwestern University, Glendale, AZ USA; 2 Center for Health Promotion and Disease Prevention, Edson College of Nursing and Health Innovation, Arizona State University, 500 N. 3rd St, Phoenix, AZ 85004 USA; 3 Division of Pediatric Endocrinology and Diabetes, Phoenix Children’s Hospital, Phoenix, AZ USA; 4 Department of Radiology, Phoenix Children’s Hospital, Phoenix, AZ USA; 5 Division of Gastroenterology, Phoenix Children’s Hospital, Phoenix, AZ USA; 6 Department of Child Health, University of Arizona College of Medicine, Phoenix, AZ USA; 7 Office of Research, Phoenix Children’s Hospital, Phoenix, AZ USA; 8 Division of Pediatric Critical Care Medicine, Phoenix Children’s Hospital, Phoenix, AZ USA; 9 Southwest Interdisciplinary Research Center, Arizona State University, Phoenix, AZ USA

## Abstract

**Background:**

The application of advanced imaging in pediatric research trials introduces the challenge of how to effectively handle and communicate incidental and reportable findings. This challenge is amplified in underserved populations that experience disparities in access to healthcare as recommendations for follow-up care may be difficult to coordinate. Therefore, the purpose of the present report is to describe the process for identifying and communicating findings from a research MRI to low-income Latino children and families.

**Methods:**

Latino adolescents (*n* = 86) aged 12–16 years old with obesity and prediabetes underwent a research MRI (3 Tesla Philips Ingenia®) as part of a randomized controlled diabetes prevention trial. The research MRIs were performed at baseline and 6 months to assess changes in whole-abdominal fat distribution and organ fat in response to the intervention. An institutional pathway was developed for identifying and reporting findings to participants and families. The pathway was developed through a collaborative process with hospital administration, research compliance, radiology, and the research team. All research images were reviewed by a board-certified pediatric radiologist who conveyed findings to the study pediatrician for determination of clinical actionability and reportability to children and families. Pediatric sub-specialists were consulted as necessary and a primary care practitioner (PCP) from a free community health clinic agreed to receive referrals for uninsured participants.

**Results:**

A total of 139 images (86 pre- and 53 post-intervention) were reviewed with 31 findings identified and 23 deemed clinically actionable and reportable. The only reportable finding was severely elevated liver fat (> 10%, *n* = 14) with the most common and concerning incidental findings being horseshoe kidney (n = 1) and lung lesion (n = 1). The remainder (*n* = 7) were less serious. Of youth with a reportable or incidental finding, 18 had a PCP but only 7 scheduled a follow-up appointment. Seven participants without a PCP were referred to a safety-net clinic for follow-up.

**Conclusions:**

With the increased utilization of high-resolution imaging in pediatric research, additional standardization is needed on what, when, and how to return incidental and reportable findings to participants, particularly among historically underrepresented populations that may be underserved in the community.

**Trial registration:**

Preventing Diabetes in Latino Youth, NCT02615353

## Background

Disclosing research results to study participants remains controversial as researchers must balance the ethical obligation of the participant’s right to know with the uncertainty of the benefit or risk of knowing such information [1]. Incidental findings (IFs) add further disclosure challenges for researchers as they often represent a discovery unrelated to the purpose of the study but may be valid and medically actionable [2, 3]. IFs are common with the application of state-of-the-art imaging techniques in clinical research [4], yet there is no consensus on a systematic process for when, how, and to whom IFs should be communicated back to participants. Governing bodies do not require or prohibit the return of IFs to participants but state that investigators should inform participants when medical care is needed for conditions or illnesses unrelated to the study or the disease being studied [2, 3, 5, 6]. However, what to do with this information, and how to effectively communicate it, especially for the non-clinician researcher, underscores the importance of collaboration between researchers and practitioners [6] that has implications that span medicine, ethics, law, and public health.

Communication of IFs may be further complicated among underserved and otherwise underrepresented pediatric populations who experience a disproportionate burden of disease due to social determinants of health [7, 8]. For example, an IF that warrants follow up care in low-income research participants may present additional challenges due to factors such as limited access to primary and specialty care [9–14], challenges navigating the healthcare system [15–17], and/or low health literacy [18–20]. As efforts are made to increase the diversity of clinical trial populations by enrolling underrepresented and underserved groups [21], it is critical for researchers to consider contextual and structural factors that are operational within socially disadvantaged populations so that research participation does not exacerbate existing heath disparities.

There is an extensive body of literature discussing IF identification during imaging research studies among adults [22–24]. In contrast, this topic has only recently received attention in children with most research focused on brain imaging studies [25–28]. High resolution imaging techniques to measure ectopic fat depots among youth has gained traction to better understand the current epidemic of pediatric obesity and related conditions [29–31]. The increased use of advanced imaging techniques in pediatric obesity research coupled with the existing disparities in obesity and related conditions make it likely that IFs will be identified in participants that may experience barriers to following up on such findings. However, there are no published pathways or protocols for handling IFs in the pediatric obesity research literature.

Therefore, the aims of the current report are: 1) describe the process for identifying and returning findings from a research MRI to low-income Latino children and families participating in a randomized clinical trial with an imaging component, 2) characterize the number, type, and potential implications of findings on follow-up management, and 3) present key lessons learned throughout the process.

## Methods

### Subjects

As part of the Preventing Diabetes in Latino Youth study, a randomized controlled trial evaluating the efficacy of a lifestyle intervention for the prevention of type 2 diabetes mellitus (T2D) among Latino youth with obesity and prediabetes, youth and their families were offered the opportunity to undergo a research MRI for assessment of regional fat distribution including subcutaneous abdominal adipose tissue, visceral adipose tissue, pancreatic fat, and liver fat. The current report describes the process for identifying and communicating findings discovered during the research MRI along with the number, type, and follow-up recommendations to families.

**Fig. 1:**
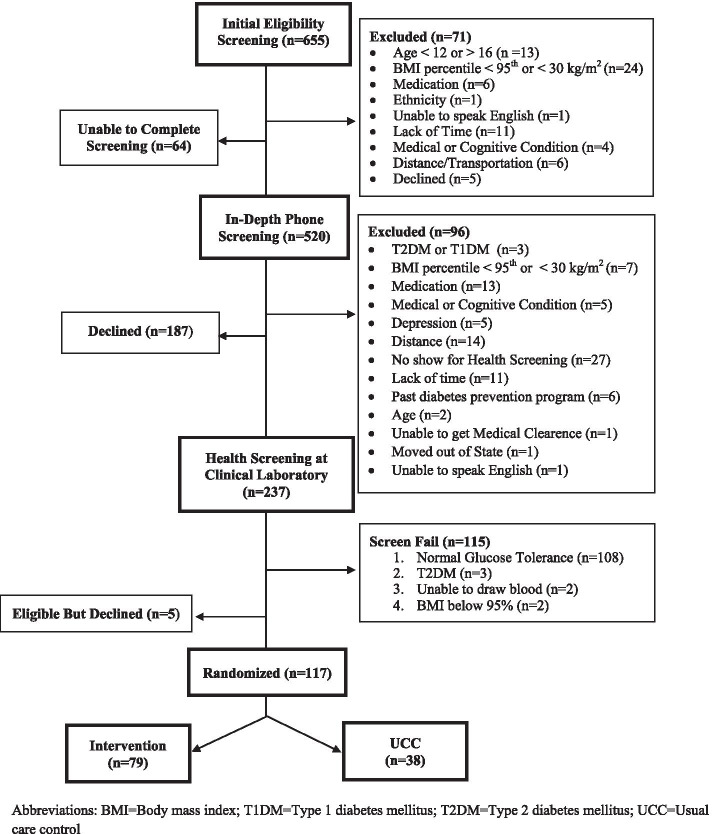
Preventing Diabetes in Latino Youth CONSORT Flow Diagram

Details of the Preventing Diabetes in Latino Youth study design and methodology have been previously published [32]. Participants were enrolled from a variety of community and clinical settings [33] and randomized to either six-month lifestyle intervention or a usual care control group. Inclusion criteria were youth aged 12–16 years old at enrollment, who self-identified as Latino, with obesity (BMI ≥95th percentile for age and sex or a BMI ≥30 kg/m^2^), and prediabetes (hemoglobin A1c between 5.7–6.4%, fasting glucose between 100 and 125 mg/dL, or 2-h glucose between 120 and 199 mg/dL). Figure 1 summarizes youth screened, excluded, and included for the parent study. This study was approved by both Arizona State University’s and Phoenix Children’s Hospital Institutional Review Boards. Informed consent was obtained from a parent/guardian and assent was obtained from all youth prior to undergoing research related MRIs. During the consent process, the youth and parent/legally authorized representative were informed of the potential discovery of unrelated incidental or reportable findings and if warranted would be notified of any such finding.

### MR imaging acquisition

All MRI studies were acquired on the same 3 T scanner (Philips Ingenia 3 T MRI), with a 44-channel posterior table coil, located in the Department of Radiology at Phoenix Children’s Hospital. All scans were conducted by a board-certified MR imaging technologist by use of a standard protocol. A single-breathhold 6-echo acquisition with a 7 peak fat modeling, and T2* correction to create 3D fat fraction maps. Parameters used were TE:12/16/20/24 ms; TR:7.68 ms; T1, 18 ms; FOV, 400 × 400 mm; effective voxel resolution, 1X1X1 mm^3^; scanning time, 5 min.

### Development of institutional pathway for identification and reporting of incidental findings

**Fig. 2:**
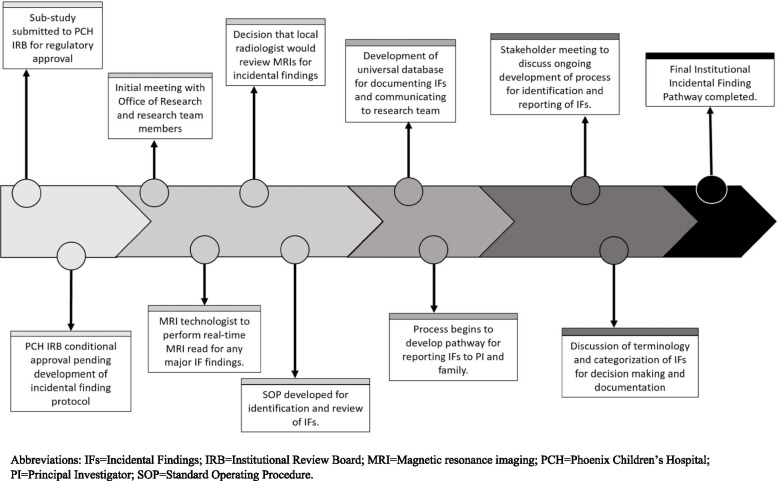
Timeline of Major Events Leading to Development of an Institutional Pathway

An iterative process that engaged hospital administration, research compliance, radiology, and the research team was used to develop a pathway for identifying and reporting findings to participants and families. Figure 2 provides a timeline of major events, meetings, and decisions that contributed to the development of the final pathway.

### Assessment and classification of incidental findings

All MR images were reviewed by a board-certified pediatric radiologist who communicated findings to the project physician (i.e., a pediatric endocrinologist) for additional consideration. The project physician evaluated each finding to determine reportability and deemed findings incidental if they were medically important, clinically actionable, and beyond the scope of the study; and reportable if they were medically important and clinically actionable.

Because the research was conducted at a tertiary care center in a children’s hospital, several pediatric sub-specialists were available for consultation in the determination of whether a finding was reportable and, if so, what additional clinical actions may be recommended. Once deemed clinically actionable, the project physician communicated the finding and provided recommendations for follow-up care to the participant and their parent / legally authorized representative(s) via telephone and in writing. Whenever appropriate, written communication was translated into Spanish and verbal communication was performed with Spanish interpreters or Spanish speaking physicians. Since youth were recruited from a variety of settings, findings were only discussed with the participant’s primary care provider (PCP) if the family provided consent for a release of records. If the family provided consent, the project physician provided the PCP with both verbal and written communication regarding the finding including any recommended next steps. Participants without insurance were referred to a free community health clinic where the medical director, a PCP, agreed to receive referrals and help coordinate follow-up as necessary.

## Results

### Participant demographics

Table 1 summarizes demographic data, including anthropometric and socioeconomic data, of participants. Parental education level varied; however, the majority of parents had a high school education or less (71%, *n* = 61). Almost 80% (*n* = 68) of participants were covered under the Arizona Medicaid Program for health insurance and 51% (*n* = 44) participated in a federal food assistance program including Supplemental Nutrition Assistance Program (SNAP), SNAP for Women, Infants, and Children (WIC), or a combination of both.

**Table 1:** Demographic, Anthropometric, and Socioeconomic Characteristic of Participants (*n* = 86) in Imaging Substudy

Variable	Mean ± SD or % (n)
Age (years), mean ± SD	13.6 ± 1.4
Gender, % male (n)	60.5% (52)
BMI (kg/m^2^), mean ± SD	34.1 ± 5.4
BMI Percentile (%), mean ± SD	98.5 ± 1.2
Recruitment Site, % (n)	
*Clinical*	51.2% (44)
*Community*	17.4% (15)
*Media*	23.3% (20)
*Word of Mouth*	8.1% (7)
Parent Highest Education Level, % (n)	
*Less than 6th grade*	4.7% (4)
*Completed elementary school (6th grade)*	22.1% (19)
*Completed middle school (9th grade)*	25.6% (22)
*Completed high school (12th grade)*	18.6% (16)
*Completed technical school*	8.1% (7)
*Some college*	10.6% (9)
*College graduate or higher*	7.0% (6)
Parent Income Level, % (n)^a^	
*$0 to $500 per month*	2.3% (2)
*$501 to $1000 per month*	8.1% (7)
*$1001 to $2000 per month*	10.5% (9)
*$2001 to $3000 per month*	8.1% (7)
*$3001 to $4000 per month*	1.2% (1)
Household size (total # of people), mean ± SD	5.3 ± 1.7
Government Assistance, % yes (n)	
*WIC*	18.6% (16)
*State Medicaid Program*	79.1% (68)
*Food Stamps (Nutrition Assistance Program)*	40.7% (35)
*Section 8 Voucher*	1.2% (1)
*Disability Insurance*	7.0% (6)
*Other type of government assistance*	1.2% (1)

Abbreviations: BMI=Body mass index; SD=Standard deviation; WIC=Special Supplemental Nutrition Program for Women, Infant, and Children

^a^Parental income level had 60 individuals indicating do not know or refused to answer

### Institutional pathway for identification and reporting of incidental findings

**Fig. 3:**
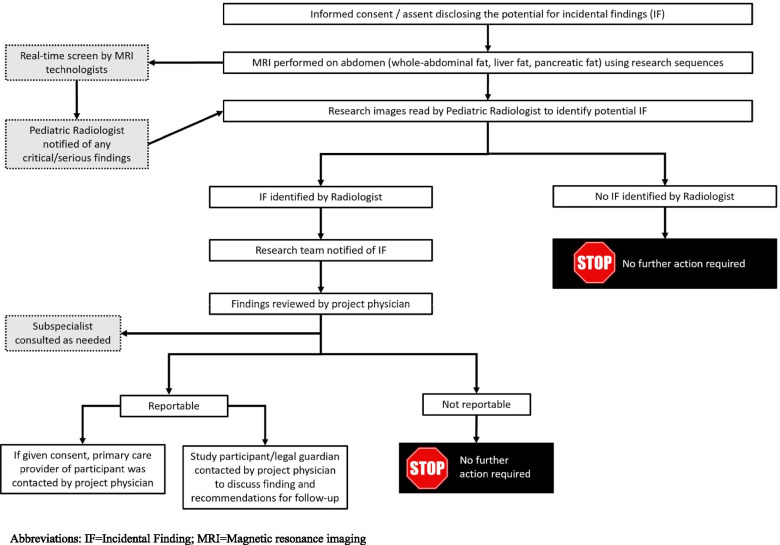
Institutional Pathway for Identification and Reporting Incidental (IFs) and Reportable Findings

The iterative process outlined in Fig. 2 resulted in the development and implementation of an institutional pathway (Fig. 3) for the identification, categorization, and communication of findings discovered during a research MRI.

### Incidental finding prevalence

MRIs were performed on 86 youth at baseline and 53 post-intervention yielding a total of 139 images. From these images, 31 findings in 25 participants were identified by the radiologist. Twenty-three of the 31 findings (75%) were deemed incidental or reportable by the project physician and communicated to youth and their families. Table 2 provides a summary of the findings among the study cohort along with national prevalence rates, clinical recommendations, and potential implications. The only reportable finding was severely elevated liver fat (*n* = 14) which was considered reportable if found to be > 10%. The most concerning IFs were horseshoe kidney (n = 1) and a lung lesion (n = 1) while the remainder (*n* = 7) were considered less serious.

**Table 2:** Reportable and Incidental Findings for Study Cohort with National Prevalence, Recommendations, and Potential Implications of Condition

Condition	Study Proportion	Population Prevalence	Recommendations	Implication and Differential Diagnosis
Cholelithiasis	2.3% (2)	1.9–4.0% [34]*	• Evaluation by PCP. • If child develops abdominal pain, vomiting, or fever, please go to the ED.	• No evidence of inflammation most likely indicates no acute health problems.
Horseshoe kidney	1.2% (1)	0.16% [35]*	• Evaluation by PCP. • Referral to Pediatric Nephrologist.	• Increased risk for hydronephrosis, urinary obstruction, renal calculi, and urinary tract infections.
Left lung lesion	1.2% (1)	Not applicable	• Evaluation by PCP. • Referral to Pediatric Pulmonologist.	• Possible causes include pneumonia, granulomatous disease, and metastatic cancer.
Hepatic cyst	1.2% (1)	2.5% [36]	• Evaluation by PCP. • Referral to Pediatric Gastroenterologist. • Liver ultrasound.	• Most likely benign, however other possible causes include neoplasm, abscess, and hemangioma.
Renal cyst	2.3% (2)	0.1–0.25% (ADPKD) [37]* 0.003–0.01% (ARPKD) [37]*	• Evaluation by PCP. • Referral to Pediatric Nephrologist. • Renal ultrasound.	• Most likely benign, however may represent congenital or acquired polycystic kidney disease.
Ovarian cyst(s)	2.3% (2)	6–20% [38]	• Evaluation by PCP.	• Most likely benign and will resolve without intervention. • May be suggestive of polycystic ovarian syndrome.
Severely elevated liver fat > 10%^a,b^	16.3% (14)	9.6% [39]*	• Evaluation by PCP. • Referral to Pediatric Gastroenterologist.	• Most likely etiology is nonalcoholic fatty liver disease, however, cannot rule out other potential causes of liver fat.

Abbreviations: ADPKD = Autosomal Dominant Polycystic Kidney Disease; ARPKD = Autosomal Recessive Polycystic Kidney Disease; ED = Emergency Department; PCP = Primary Care Physician

*Asterisk indicates the population prevalence is specific to pediatrics

^a^ This is based on prevalence of non-alcoholic fatty liver disease (NAFLD) as defined by liver fat > 5.5%. NAFLD prevalence increases among Hispanic youth (11.8%) and youth with obesity (38%)

^b^ Severely elevated liver fat was deemed a reportable finding based upon discussions with the IRB

### Communication of IF to youth, family, and PCP

Of the 25 participants found to have either an incidental or reportable finding, 18 (72%) had a PCP but only seven (29%) followed-up with their PCP to discuss the IF. Participants that did not have a PCP (n = 7) were referred to the community clinic that provides care for uninsured patients.

## Discussion

The percentage of youth with MRI findings in our study (29%) was higher than previous studies with a range of 11–27% [25, 26, 28, 40, 41]. In addition, the majority (75%) of findings in the current study met the internal threshold for reportability with 39% deemed incidental, which was substantially greater than previous reports (range: 0.5–36%) [25, 26, 28, 40, 41]. There were key lessons learned throughout the process that aided the development and implementation of the institutional pathway with the key takeaway being the need to better understand and assess the benefits as well as the potential harms associated with communicating research MRI findings. This is of particular importance among underrepresented populations where other challenges emerge such as low health literacy and barriers to follow-up care. By providing a detailed description of the process implemented at our institution, we hope to provide valuable information needed for building a broader consensus and unified management protocol for reporting IFs while considering social determinants of health.

Treatment protocols for the management of IFs for nondiagnostic MRI have been previously published among adult populations [23] or neuro-imaging studies [24–26, 28, 40, 41]. The published protocols for the identification, classification, and communication of IFs among pediatric populations [28, 40, 42], highlight the importance of explaining risks and benefits of IFs during the consent process, incorporation of multiple expert opinions in documenting and categorization of IFs, and the communication of IFs to participants [28, 40]. However, previously published pediatric protocols for IFs were established for brain MRIs in otherwise healthy children [28, 40], which is in contrast to the current study where youth with obesity and prediabetes underwent an abdominal MRI. Not surprisingly, the likelihood of discovering an IF differs by region of interest [23, 43, 44] and with the use of more sophisticated imaging platforms [25]. Previous studies have reported an IF prevalence of 36% among adults who underwent a whole-body research MRI [23]; unfortunately, there are no published pediatric prevalence rates for whole-body or abdominal MRIs performed during pediatric research. However, a recent systematic review of pediatric whole-body MRI scans for diagnostic purposes [43] found only one study which reported IFs prevalence of 96% (*n* = 55) [44]. The relatively high prevalence of findings in the current study may be largely driven by the inclusion of fatty liver as a reportable finding. Given that liver fat was an a priori outcome of interest in the parent trial and Latino youth with obesity are at increased risk for fatty liver [45–47], it was more appropriate to consider elevated liver fat as a reportable finding rather and an IF. Discussions with the IRB and pediatric hepatologists at our institution led to the decision to report elevated liver fat to families. However, rather than using the current clinical recommendation of > 5% liver fat [48], we applied a more conservative threshold of > 10% at 6-months as reportable. The rationale being that the intervention under investigation (lifestyle behavior change) is considered a first line treatment for pediatric fatty liver disease but in the absence of improvement, and at a level two-fold greater than normal, warrants additional consideration and work-up. If we omit fatty liver as a reportable, the cumulative prevalence for both reportable and incidental findings in the current study is 29%. Definitions notwithstanding, the enhanced utilization of new technologies increase the likelihood of primary and IF discovery that warrant follow-up; therefore, additional guidance and standardization on how best to handle IF outside of clinical care is needed.

In deciding whether to deem a finding reportable, the research team must make a subjective decision in determining the clinical significance and actionability of a finding. The research team needs to first weigh the importance of an IF along a spectrum of clinical significance [49]. However, the MRI performed for research purposes in many cases cannot determine where the IF falls on the spectrum. Even after deeming a finding clinically relevant, the research team is challenged with effectively communicating the medical importance of an IF to multiple individuals (i.e., youth, family, and PCP) when its clinical significance is unclear. This is further complicated when working with participants from a low-income, health disparate population [9, 10].

Differences in culture, language, health literacy, and access to care have the potential to magnify the inherent challenges faced in communicating the clinical significance of findings. Although we did not assess health literacy, the majority (64%, *n* = 78) of the primarily Spanish-speaking parents in the study completed at least 9th grade. Parental health literacy is intimately tied with child health [50]; however, research that prioritizes underserved adolescent populations typically encounter youth that are of higher health literacy than their parents [51, 52], or have the technological know-how to seek out additional information [53]. Therefore, it stands to reason that returning IFs in populations with low health literacy warrants additional considerations to further minimize undue worry and confusion.

Research that prioritizes vulnerable and underserved populations with limited access to care may amplify both the benefit and harm associated with the discovery and communication of IFs. Understanding that the communication of IFs may lead to additional costs and follow-up care, but most importantly, unnecessary anxiety and stress needs not only be considered but also adequately assessed in imaging research. With the intention to diversify study populations within clinical research trials [54], it may be warranted to allocate additional funds or resources for care beyond the scope of the study in order to prevent greater health disparities [9, 10] in an already at-risk population. Social determinants of health exacerbate equitable access to care in an already fragmented healthcare system. As researchers we need to challenge ourselves to address not only the complexities of communicating uncertain findings to research participants but also how clinical research can address social determinants of health.

There were several lessons learned during the process of developing and implementing an institutional pathway for the identification and communication of incidental and reportable findings during nondiagnostic abdominal MRIs. These lessons learned have been compiled into recommendations and considerations for addressing findings discovered during pediatric research (Table 3). Through the entire process, the most prominent takeaway was the need to better understand the risk-to-benefit ratio of reporting findings to ensure potential repercussions are minimized for youth, families, and the PCP. The communication of findings must include a benefit [55] such as early identification of a clinically relevant health condition that leads to early prevention and treatment or equipping research participants with new information that enables them to make more informed decisions [1, 56]. These possible benefits must be weighed against the potential cost of unnecessary medical visits, diagnostic studies, and treatments; as well as the anxiety induced for participants and their medical providers due to the incomplete, unclear significance of a given finding.

**Table 3:** Recommendations and Considerations for Addressing Incidental and Reportable Findings in Pediatric Research

Area.	Recommendation
Funding agencies	Allocate fund use when an incidental finding is discovered to assist in provision of follow-up care, particularly among underserved populations that lack access to specialty care and/or insurance.
Scientific Organizations	Establish best practices and scientific statements to guide pediatric researchers who encounter incidental findings during investigations
Informed Consent	Clearly explain the nondiagnostic intent of the MRI as well as the possibility of discovering unrelated, unintended but potentially clinically relevant findings.
Study design	Track the impact of incidental finding communication to the participant and their family, including additional clinical visits and diagnostic tests, to better assess the financial and time-related costs and potential health benefits.
Methodology	Develop a process for how incidental findings will be identified and communicated among members of the research team as well as to families and primary care providers.

Our study had several limitations. First, all youth were of Latino ethnicity which limits the generalizability to other populations. Second, we did not assess the health literacy of the youth and their parent/guardians which may impact the effectiveness and cost associated with communicating uncertain and incomplete medical information. Third, the study was not designed to prospectively evaluate the impact of IFs on future outcomes and participants were not randomized based upon receiving or not receiving findings. Lastly, we did not attempt to quantify the cost to the families due to the IF or the long-term implications, which represents a gap in the literature. The ability to accurately assess the risk-to-benefit ratio of communicating IF includes evaluation of the financial cost, time and productivity lost, and anxiety that results from this process. This is even more important among populations with health disparities where differences in cultural values, language barriers, and other social determinants of health increase the possibility of miscommunication leading to greater confusion and anxiety. Despite these limitations, there were several strengths to our study including that one pediatric radiologist read all the images reducing variability in reporting. Additionally, a single high-field scanner with high-resolution imaging was used for all participants providing a benchmark for the prevalence of incidental findings among a homogenous but underrepresented cohort of Latino youth with obesity.

## Conclusion

To our knowledge, this is the first study among a pediatric population that has described prevalence rates and a protocol for reporting incidental and reportable findings discovered during a nondiagnostic abdominal MRI. As clinical trials increasingly use high-field, high-resolution magnets, the establishment of single institution protocols and pathways are important steps towards building broader consensus for handling such findings. Most importantly is the need to adequately weigh the potential benefits with the associated risks of communicating uncertain, incomplete medical information. We hope that our experience will empower other researchers to consider the sociocultural context of study populations when developing protocols and encourage imaging studies to track the financial and psychosocial implications associated with communicating incidental and reportable findings, particularly among underserved and underrepresented populations.

## Data Availability

The datasets used and/or analyzed during the current study are available from the corresponding author on reasonable request.
